# The HIRA Complex Subunit Hip3 Plays Important Roles in the Silencing of Meiosis-Specific Genes in *Schizosaccharomyces pombe*

**DOI:** 10.1371/journal.pone.0019442

**Published:** 2011-04-29

**Authors:** Fumitaka Mizuki, Aki Tanaka, Yutaka Hirose, Yoshiaki Ohkuma

**Affiliations:** Laboratory of Gene Regulation, Graduate School of Medicine and Pharmaceutical Sciences, University of Toyama, Toyama, Japan; Institute of Genetics and Molecular and Cellular Biology, France

## Abstract

**Background:**

The control of gene expression is essential for growth and responses to environmental changes in various organisms. It is known that some meiosis-specific genes are silenced during mitosis and expressed upon nitrogen starvation in *Schizosaccharomyces pombe.* When the factors responsible for this regulation were studied, a *hip3* mutant was isolated via discovery of a defect in the transcriptional repression of meiosis-specific genes. Hip3 is a subunit of the HIRA (histone regulatory complex A) complex, which consists of four subunits (Hip1, Hip3, Hip4 and Slm9) and acts as a histone chaperone that is independent of DNA replication.

**Methodology/Principal Findings:**

In a search for mutants, the meiosis-specific gene *SPCC663.14c* ^+^ was identified by screening for genes that are silenced during mitosis and induced upon nitrogen starvation. A reporter plasmid that expresses the *ura4* ^+^ gene driven by the *SPCC663.14c* ^+^ promoter was constructed. Screening for suppressor mutants was then carried out in nitrogen-rich medium without uracil. A mutant with a mutation in the *hip3^+^* gene was isolated and named *hip3-1*. This mutation alleviated the transcriptional repression of the *ura4^+^* gene on the reporter plasmid and of the endogenous *SPCC663.14c* ^+^ gene in the presence of nitrogen. A ChIP assay revealed that RNA polymerase II (Pol II) and TFIIE were enriched at the *SPCC663.14c* ^+^ locus, whereas the levels of histone H3 were decreased in *hip3-1* cells. Intriguingly, histone H3 was heavily modified at the *SPCC663.14c* ^+^ locus in *hip3-1* cells; these modifications included tri-methylation and acetylation of H3 lysine 9 (H3K9), mono-methylation of H3 arginine 2 (H3R2), and tri-methylation of H3 lysine 4 (H3K4). In addition, the tri-methylation of H3K9 and H3K4 were strongly elevated in *hip3-1* mutants.

**Conclusions:**

Taken together, these results indicate that Hip3 plays important roles in the control of histone modifications at meiosis-specific gene loci and induces their transcriptional repression.

## Introduction

In eukaryotes, genomic information is hierarchically compacted into chromatin. Thus, it is necessary to open the chromatin structure upon the stimulation of gene expression [1], [2]. The regulation of gene expression is required for proper growth responses to environmental changes [3], [4]. Transcription is the first, and therefore most critical, step in gene expression. In eukaryotes, RNA polymerase II (Pol II) catalyzes the transcription of all the protein-coding genes. Pol II and five general transcription factors (TFIIB, -D, -E, -F and -H) form a preinitiation complex (PIC) on promoter DNA [5], [6]. The Mediator complex is a large complex that consists of approximately 30 subunits and bridges transcriptional regulatory factors and the PIC to transduce transcriptional signals to the PIC [7]. It is now known that Mediator recruits chromatin-remodeling complexes and histone-modifying enzymes in addition to transcriptional cofactors. Chromatin is coordinately regulated by Pol II during transcription.

Chromatin consists of nucleosomes with histone octamers (H2A, H2B, H3 and H4). This chromatin structure is highly conserved among eukaryotes. To date, various enzymes have been identified as modifying the N-terminal region of each histone protein via processes such as acetylation, methylation, ubiquitination, sumoylation, and phosphorylation [8], [9]. These modifications are coupled to chromatin processes, including replication, recombination, DNA repair and transcription [10]. It is known that many genes respond to various environmental changes, including changes in temperature and starvation. In the fission yeast *Schizosaccharomyces pombe* (*S. pombe*), more than one hundred genes are activated under starvation conditions [11], [12]. Starvation induces meiosis, in which two haploid cells fuse and form a diploid cell to generate four spores.

Meiosis is a key step in the generation of genetically individual cells. In *Schizosaccharomyces pombe*, haploid cells are divided into two mating types: *h^-^* and *h^+^*. Haploid cells of opposite mating types are fused to form a diploid cell and immediately undergo meiosis and sporulation. Nutritional starvation induces expression of the *ste11* ^+^ gene, which encodes a DNA-binding protein belonging to the high-mobility group (HMG) family and binds to a specific DNA sequence (TTCTTTGTTY) called the TR box [13]. Ste11 is a transcription factor that plays a role in the expression of most meiosis-specific proteins, including an RNA binding protein Mei2 involved in meiosis regulation [13]. Thus, Ste11 is a key activator of meiosis [14]. Because meiosis-specific genes are usually repressed in rich medium, regulating Ste11 expression is important. The cAMP-Pka1 pathway has been reported to regulate the onset of differentiation primarily by repressing the *ste11* ^+^ gene [15]. In this pathway, starvation leads to a decrease in the cellular cAMP level and activates the transcription factor Rts2, which directly controls *ste11* ^+^ transcription. Although it is now widely accepted that many proteins are involved in gene silencing, their functional mechanisms are still elusive [16].

To understand the transcriptional repression mechanisms of meiosis-specific genes using vegetative fission yeast cells, we constructed a plasmid containing the *ura4^+^* gene driven by a meiosis-specific gene promoter and isolated mutants that expressed the *ura4^+^* gene when grown on plates containing a nitrogen source. One of the positively screened mutants had a mutation in the *hip3^+^* gene and was named *hip3-1*. Hip3 is a component of the HIRA complex, which is known to function as a histone chaperone that is independent of DNA synthesis [17], [18]. *hip3-1* cells were found to exhibit a temperature-sensitive (*ts^−^*) phenotype. The *hip3-1* mutant protein (Hip3-1) is truncated and lacks the C-terminus, which contains a tetratricopeptide repeat (TPR) motif. A ChIP assay revealed that Pol II is located at the *SPCC663.14c* ^+^ locus in *hip3-1* mutants but not in wild-type cells. Interestingly, acetylated H3K9 was also found to be enriched at this locus in *hip3-1* mutants. These data suggest that the HIRA complex is involved in both chaperoning histones to DNA and in histone modification, and that this complex ultimately plays a role in cell-cycle regulation.

## Results

### Isolation of Suppressor Mutants Involved in Transcriptional Repression of Meiosis-specific Genes

**Figure 1:**
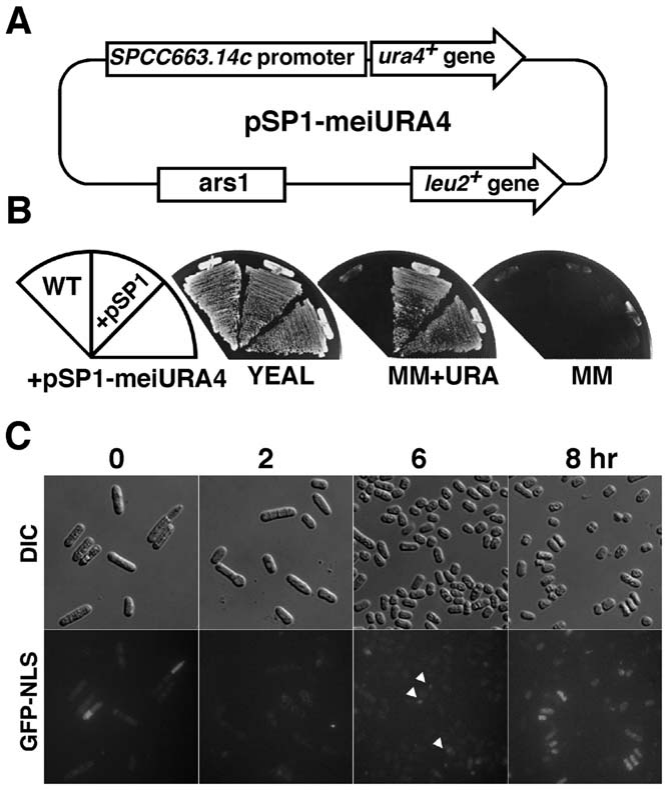
Construction of the reporter plasmid. (A) The structure of the pSP1-meiURA4 reporter plasmid. The intergenic region between *SPCC663.14c* ^+^ and *SPCC663.15c* ^+^ and the *URA4^+^* ORF was amplified by PCR. These fragments were inserted into the vector pSP1. (B) Repression of the *URA4^+^* gene from the *SPCC663.14c* ^+^ promoter. UR471 cells were transformed with the pSP1 vector or the pSP1-meiURA4 plasmid and streaked onto YEAL, MM+uracil or MM plates. (C) The kinetics of GFP-NLS induction at the *SPCC663.14c* ^+^ promoter. pSP1-meiGFP-NLS transformants were incubated in MM until mid-log phase. After washing with water, the cells were suspended in MM-N medium for the indicated times.

To isolate *S. pombe* mutants that alleviate transcriptional silencing of meiosis-specific genes, we constructed a reporter plasmid (pSP1-meiURA4) expressing the *ura4* ^+^ gene driven by various meiosis-specific gene promoters (Fig. 1A). *SPCC663.14c* ^+^ was identified as a meiosis-specific gene by screening for genes that are repressed during mitosis and induced upon nitrogen starvation according to the *S. pombe* gene DB (http://old.genedb.org/genedb/pombe/) [19]. *leu* ^−^ *ura* ^−^ cells transformed with the pSP1-meiURA4 plasmid grew on minimal medium (MM) plates containing uracil but not on plates without uracil. These results indicate that *ura4^+^* gene transcription was silenced (Fig. 1B). To determine whether the *SPCC663.14c* ^+^ gene promoter is activated during nutritional starvation, we modified pSP1-meiURA4 by replacing the *ura4* ^+^ gene with a gene encoding a GFP-NLS fusion protein (pSP1-meiGFP-NLS). pSP1-meiGFP-NLS transformants were then incubated under nutritional starvation conditions (MM-N) for various lengths of time. The pSP1-meiGFP-NLS transformants did not express GFP-NLS under normal conditions (0 hr), but GFP-NLS expression gradually increased in a time-dependent manner (Fig. 1C, arrowhead), indicating that this promoter is repressed under normal conditions and activated upon nutritional starvation. We mutagenized approximately 5 million cells containing pSP1-meiURA4 by UV irradiation and isolated 8 *ura* ^+^ mutants that grew on MM plates. Of those, one mutant's growth was temperature-sensitive, and this mutant was therefore chosen for further analysis.

**Figure 2:**
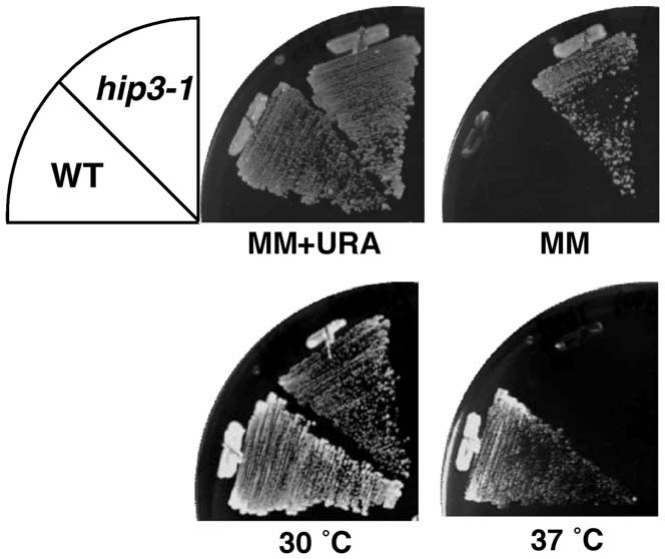
The *hip3-1* mutant has a defect in transcriptional repression and is temperature-sensitive. UR471 and *hip3-1* cells were transformed with the pSP1-meiURA4 plasmid and streaked onto MM+URA, MM or YEAL plates.

To exclude the possibility that the *ura* ^+^ phenotype of the isolated mutants resulted from mutations within the plasmid, we removed the plasmid from the mutant and then re-transfected pSP1-meiURA4. As expected, mutant cells without pSP1-meiURA4 could not grow on MM plates, whereas mutant cells that were re-transformed with pSP1-meiURA4 grew on MM plates (Fig. 2), indicating that the expression of the *ura* ^+^ phenotype in the isolated mutants depends on the expression of the *ura4* ^+^ gene in the pSP1-meiURA4 plasmid. This mutant suppresses the transcriptional repression of the reporter plasmid and is temperature-sensitive at 37°C (Fig. 2). This mutant was then backcrossed three times with the wild-type strain to remove extra mutations. The heterozygous diploids with a wild-type allele grew well at the restrictive temperature, and tetrad analysis revealed 2:2 segregation of the *ts* ^−^ and *ts* ^+^ phenotypes in all cases. *ts^−^* cells transfected with pSP1-meiURA4 were found to be viable on MM plates, suggesting that the *ts* ^−^ phenotype is recessive and caused by a single mutation that correlates with the relief of the transcriptional repression of the reporter plasmid.

### Identification of the Mutated Gene

**Figure 3:**
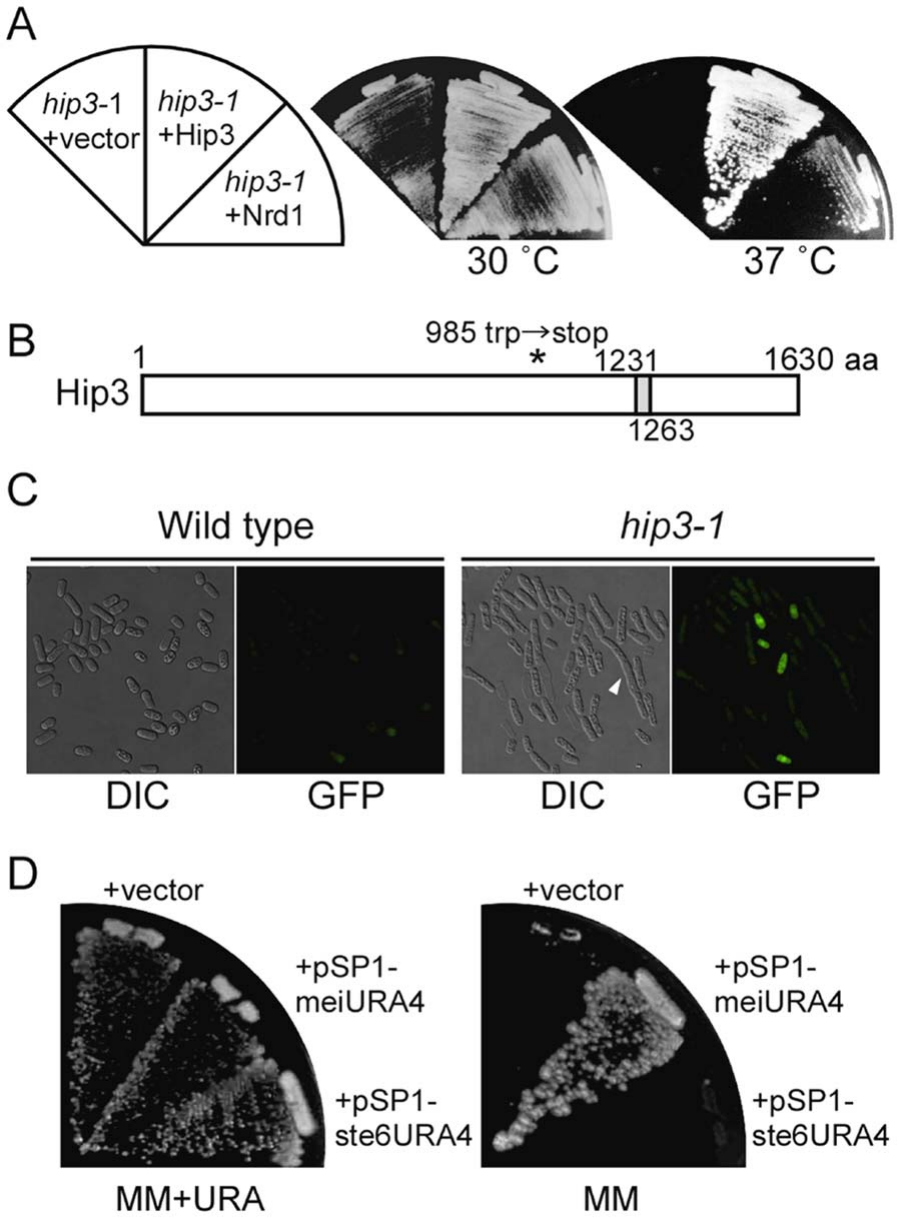
Characterization of the *hip3-1* strain. (A) The *hip3^+^* gene is responsible for the mutant phenotype. The *hip3-1* mutant was transformed with the pSP1, pTN-Hip3 or pSP1-Nrd1 plasmid and incubated on MM+uracil plates at 30°C or 37°C. (B) Schematic structure of the Hip3 protein. Hip3 has a TPR motif (gray box), and the tryptophan at amino acid 985 of the Hip3 protein is replaced with a stop codon in the *hip3-1* mutant (asterisk). (C) A defect in transcriptional repression in *hip3-1* mutants. The pSP1-meiGFP-NLS construct was transfected into UR471 and *hip3-1* cells. *hip3-1* cells have aberrant morphology (arrowhead). (D) The transcriptional repression of the *ura4^+^* gene from a meiosis-specific promoter in the *hip3-1* mutant. The pSP1, pSP1-meiURA4 or pSP1-ste6URA4 plasmid was transfected into *hip3-1* mutants and the transformants were streaked onto MM or MM+uracil plates.

To identify the gene affected by the mutation, we transformed the isolated mutant with an *S. pombe* genomic library and isolated clones that were able to complement the *ts* ^−^ growth defect. We obtained two plasmids that rescued the *ts* ^−^ phenotype. In one plasmid, a fragment containing the *nrd1^+^* gene, which encodes a protein containing an RRM (RNA recognition motif) that is involved in the transcriptional repression of STE11-regulated genes [20], was present. The other plasmid contains the *hip3^+^* gene, which encodes Hip3, a subunit of the HIRA complex. HIRA is a conserved histone chaperone that binds to *Schizosaccharomyces pombe* ASF1 (spASF1 or Cia1) to mediate replication-independent nucleosome assembly [17], [21]. The *hip3^+^* gene-containing fragment rescued the *ts* ^−^ phenotype completely, whereas the *nrd1^+^* gene-containing fragment did not rescue the *ts* ^−^ phenotype (Fig. 3A). Therefore, we presumed that the mutation was located in the *hip3^+^* gene, which was then characterized further. Sequence analysis of the *hip3* ^+^ gene revealed a single nucleotide change (G to A) at position 2954 in the protein-coding sequence that results in the replacement of tryptophan by a stop codon at amino acid residue 985 of Hip3. Thus, the isolated mutant was named *hip3-1* (Fig. 3B).

### *hip3-1* Mutant Exhibits Defects in Transcriptional Repression

To confirm that *hip3-1* mutant has a defect in the transcriptional repression of the gene driven by the *SPCC663.14c* ^+^ promoter in *hip3-1* cells, we transfected the pSP1-meiGFP-NLS plasmid into this mutant and monitored GFP-NLS expression. As expected, *hip3-1* cells expressed the GFP-NLS fusion protein in nitrogen-rich medium, but wild-type cells did not, indicating that gene silencing in the presence of a nitrogen source driven by the *SPCC663.14c* promoter is alleviated in *hip3-1* mutant (Fig. 3C). In addition, *hip3-1* cells were elongated, suggesting that Hip3 also plays an important role in cell cycle regulation (Fig. 3C, arrowhead).

Next, to investigate whether Hip3 is involved in transcriptional repression of other meiosis-specific genes, we constructed another reporter plasmid in which the *SPCC663.14c* ^+^ promoter was replaced by the *ste6* ^+^ promoter (pSP1-ste6URA4). *hip3-1* cells transformed with pSP1-ste6URA4 failed suppress the *ura^−^* phenotype (Fig. 3D), indicating that Hip3 is not involved in the transcriptional repression of all meiosis-specific genes but, rather, is specific for the *SPCC663.14c* ^+^ gene.

**Figure 4:**
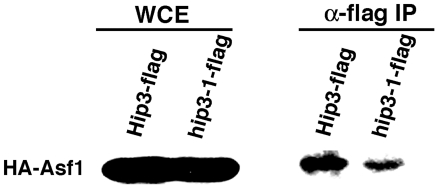
Interaction between HA-Asf1 and Hip3-1*-*flag. The pREP42-HA-Asf1 plasmid was transfected into Hip3-flag- and hip3-1-flag-expressing strains. Whole-cell extract (WCE) was immunoprecipitated using anti-FLAG M2 agarose (anti-flag IP) and analyzed by western blotting with anti-HA (3F10) and horseradish peroxidase (HRP)-conjugated anti-rat antibodies.

Hip3 is a subunit of the HIRA complex, which consists of four subunits (Hip1, Hip3, Hip4, and Slm9) and is known to interact with the histone chaperone spAsf1 (Cia1) [17], [21]. To determine whether the Hip3 mutant protein (Hip3-1) interacts with spAsf1, we carried out a co-immunoprecipitation assay (Fig. 4). The construct pREP42-HA-spAsf1, which expresses HA-tagged spAsf1 driven by the *nmt1* ^+^ promoter, was transfected into Hip3-flag- and Hip3-1-flag-expressing strains, and HA-tagged spAsf1 was induced in liquid MM medium without thiamine. After immunoprecipitation with anti-flag-M2 agarose, HA-Asf1 was detected in both strains, suggesting that the defect in transcriptional repression in *hip3-1* cells is not due to a defect in the interaction between the HIRA complex and spAsf1p.

**Figure 5:**
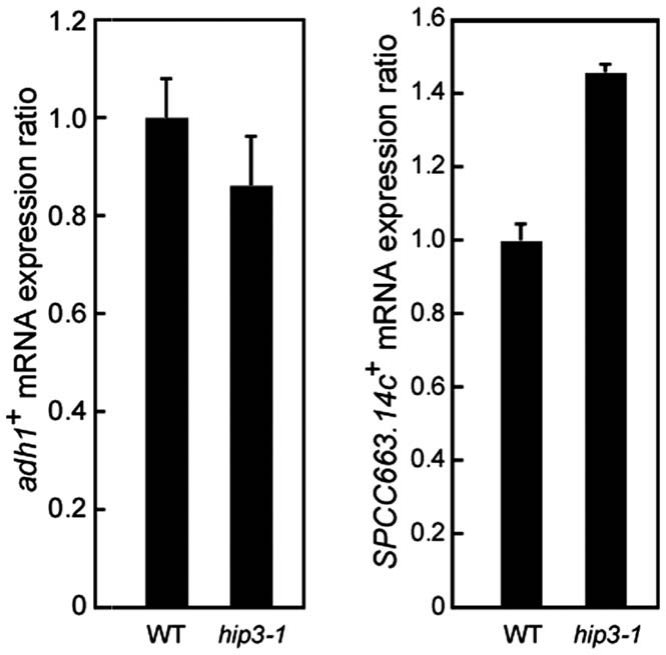
Increased levels of *SPCC663.14c* ^+^ mRNA in *hip3-1* mutants. Total RNA was prepared from wild-type and *hip3-1* cells, and RT-PCR was performed using primer sets for the indicated genes. The error bars indicate SD.

*hip3-1* mutant was isolated by screening for suppressor mutants using a reporter plasmid. To determine whether the silencing of endogenous meiosis-specific genes is defective in *hip3-1* mutant, we measured *SPCC663.14c* ^+^ transcript levels using real-time quantitative RT-PCR. We found that the level of *adh1* ^+^ mRNA was a little lower in *hip3-1* cells than in wild-type cells, whereas the level of *SPCC663.14c* ^+^ mRNA was approximately 1.4-fold higher in *hip3-1* cells than in wild-type cells (Fig. 5), suggesting that Hip3-1 produces a defect in transcriptional repression driven by the meiosis-specific gene promoters.

### *hip3-1* Mutant Exhibits Defects in Chromatin Regulation

**Figure 6:**
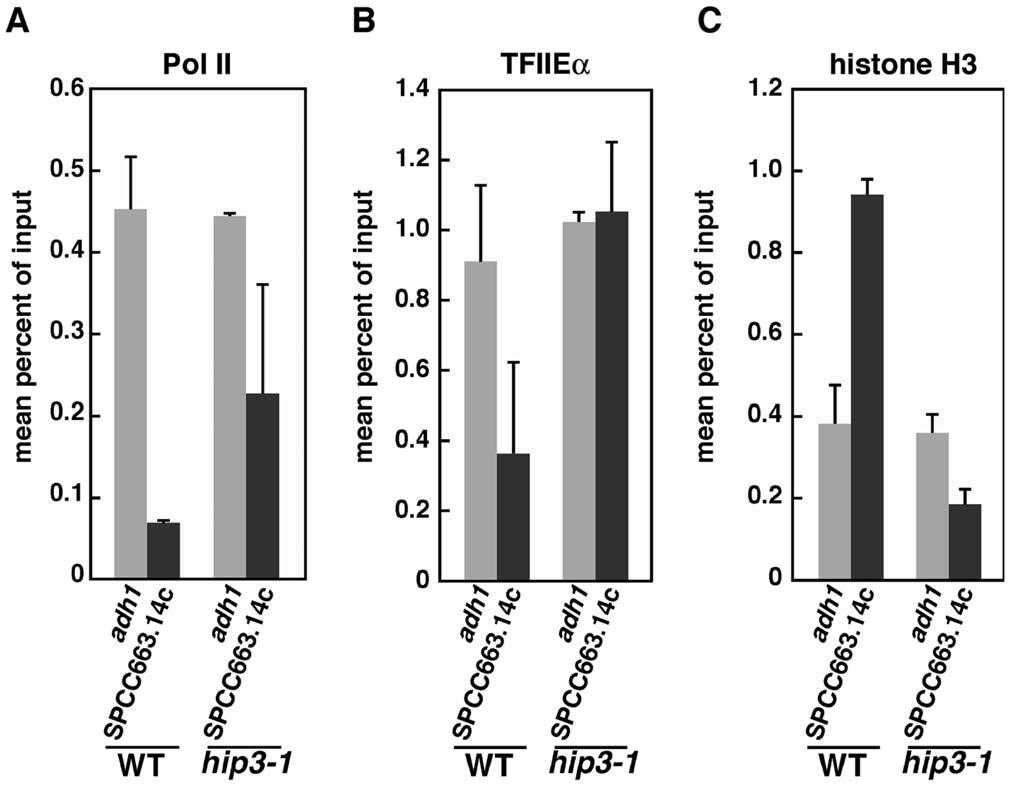
Alterations in Pol II and histone H3 enrichment at the *SPCC663.14c* ^+^ locus in *hip3-1* mutants. The indicated strains were subjected to ChIP assays using anti-spRpb3 (A), anti-spTFIIE and anti-histone H3 antibodies (C). The error bars indicate SD.

It is well known that histone chaperones play important roles in transcriptional repression by mediating nucleosome assembly and chromatin remodeling [22]. Therefore, to investigate the chromatin state at the *SPCC663.14c* ^+^ locus in *hip3-1* cells, a chromatin immunoprecipitation (ChIP) assay was carried out in wild-type and *hip3-1* cells using anti-spRpb3 (the third subunit of Pol II), anti-spTFIIEα (the large subunit of spTFIIE) and anti-histone H3 antibodies. The cells were grown in nitrogen-rich medium, and immunoprecipitations were performed using each antibody, followed by PCR using primers for the *adh1* ^+^ and *SPCC663.14c* ^+^ promoters. We found that in wild-type cells, Pol II was localized to the promoter region of the *adh1* ^+^ gene but not the *SPCC663.14c* ^+^ gene (Fig. 6A). In contrast, Pol II was localized to the promoter regions of both *adh1* ^+^ and *SPCC663.14c* ^+^ in *hip3-1* cells (Fig. 6A), suggesting that Pol II is recruited to the *SPCC663.14c^+^* promoter and engages in transcription in the *hip3-1* mutant. Furthermore, spTFIIEα was enriched at the *SPCC663.14c* ^+^ promoter region in *hip3-1* mutant cells but not in wild type (Fig. 6B). The enrichment of Pol II and spTFIIEα at this meiosis-specific gene locus suggests that the PIC forms in this region and activates *SPCC663.14c* ^+^ gene transcription. Because Hip3 is a component of the HIRA complex, we speculated that the amount of histone H3 might be lower in *hip3-1* cells than in wild type (Fig. 6C). As expected, histone H3 occupancy at the promoter region of the *adh1* ^+^ gene in *hip3-1* cells was almost the same as that in wild-type cells, whereas H3 occupancy at the promoter of the *SPCC663.14c* ^+^ gene was reduced by 5-fold in *hip3-1* cells (Fig. 6C). This result suggests that Hip3-1 has a defect in incorporating histones into histone-rich regions (such as transcriptionally repressed genes) but not into transcriptionally active genes.

**Figure 7:**
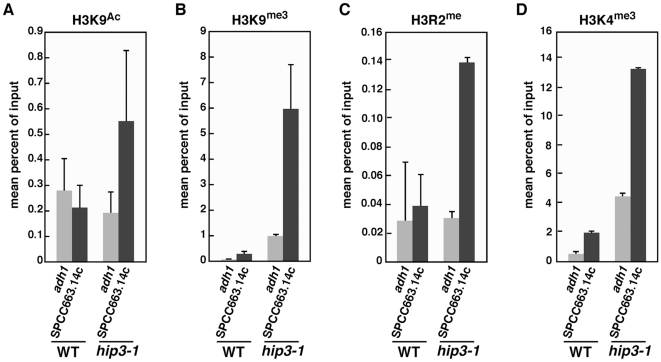
Aberrant modifications of histone H3 in *hip3-1* mutants. The indicated strains were subjected to ChIP assays using anti-acetylated H3K9 (A), anti-trimethylated H3K9 (B), mono-methylated H3R2 (C) and tri-methylated H3K4 antibodies (D). The error bars indicate SD.

Core histones (which form nucleosomes) are modified in various ways depending on their transcriptional activity. One such modification, histone H3K9 methylation, has been shown to correlate with gene silencing, whereas acetylation at the same residue correlates with transcriptional activation [23]. In *hip3-1* cells, little H3K9 acetylation occurred at the *adh1* ^+^ locus compared to wild-type cells, whereas tri-methylated H3K9 was enriched at the *adh1* ^+^ promoter (Fig. 7A). This implies that the aberrant modification of H3K9 results in a small decrease of *adh1^+^* mRNA. In *hip3-1* cells, the extent of acetylation at histone H3K9 at the *SPCC663.14c* ^+^ locus was 2.5-fold more than that in wild-type cells (Fig. 7A). Intriguingly, tri-methylated H3K9 occupancy at the *SPCC663.14c* ^+^ locus was 20-fold higher in *hip3-1* cells (Fig. 7B), suggesting that the Hip3-1 mutant has a defect in regulating the modification of histone H3 at the *SPCC663.14c* ^+^ locus, which results in the inhibition of the transcriptional repression of the *SPCC663.14c* ^+^ gene. To determine whether other histone H3 modifications are deregulated in *hip3-1* cells, we carried out ChIP assays using other modified histone H3 antibodies. H2R2 mono-methylation is a hallmark of the transcriptional activation of meiosis-specific genes in budding yeast [24], and H3K4 tri-methylation correlates with transcriptional activation and is enriched in euchromatin [25]. We found that mono-methylated H3R2 and tri-methylated H3K4 were enriched 3.5-fold and 6.5-fold, respectively, at the *SPCC663.14c* ^+^ locus in *hip3-1* cells compared to wild-type cells (Fig. 7C, 7D). These data suggest that inhibition of transcriptional repression in *hip3-1* cells is caused by unregulated histone modifications at the meiosis-specific gene *SPCC663.14c* ^+^.

### Tri-methylation of H3K9 and H3K4 are widely elevated in *hip3-1* mutants

**Figure 8:**
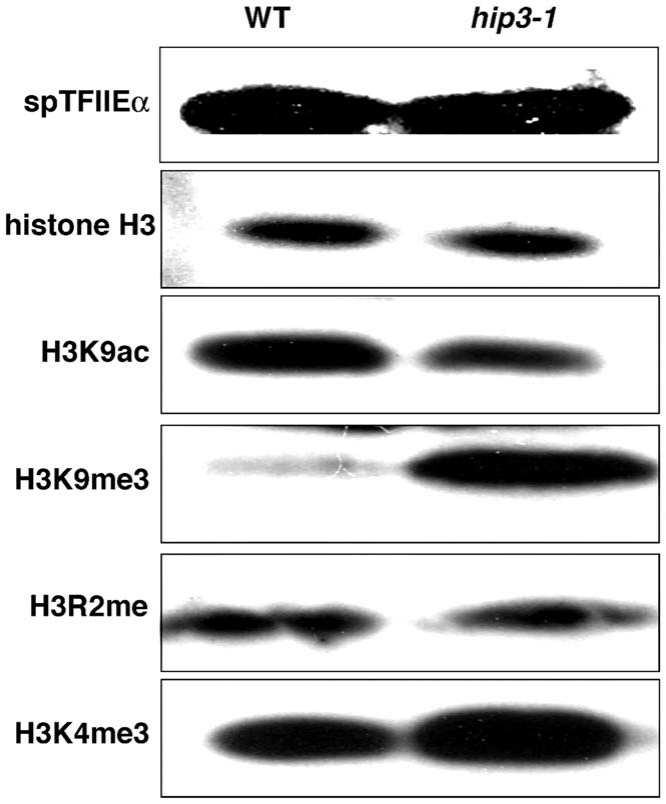
The levels of tri-methylated H3K9 are elevated in *hip3-1* mutants. Whole-cell extracts from wild-type and *hip3-1* mutant cells were analyzed by western blotting using anti-spTFIIE, anti-H3, anti-H3K9ac, anti-H3K9me3, anti-H3R2me, and anti-H3K4me3 antibodies.

In *hip3-1* mutants, several modifications of histone H3 were aberrant at the *SPCC663.14c^+^* promoter. Intriguingly, increases in tri-methylation of H3K9 and H3K4 were observed at both the *SPCC663.14c^+^* and *adh1^+^* promoters (Fig. 7). Therefore, we speculated that some of the modifications of histone H3 might be globally elevated in *hip3-1* mutants. To confirm this speculation, western blot analyses were carried out using anti-modified histone H3 antibodies, an anti-histone H3 antibody and an anti-spTFIIEα antibody as an internal control. We found that the levels of histone H3 and the mono-methylation of H3R2 were indistinguishable between wild-type and *hip3-1* mutant cells, and the acetylation of H3K9 was slightly lower in *hip3-1* cells (Fig. 8). Intriguingly, the amounts of both tri-methylated H3K9 and tri-methylated H3K4 were elevated in *hip3-1* cells (Fig. 8). Taken together, our data indicate that Hip3 contributes to the transcriptional repression of meiosis-specific genes by playing important roles in histone incorporation into chromatin and the regulation of histone modifications.

## Discussion

In this study, we isolated a mutant (named *hip3-1*) that expresses Hip3 missing its C-terminus (Hip3-1). *hip3-1* cells fail to repress meiosis-specific genes and are temperature-sensitive. We also detected defects in the embedding of histone H3 and in the control of H3 modifications at transcriptionally repressed loci in *hip3-1* mutant.

Hip3 was identified as an *S. pombe* homologue of HIR3 in *S. cerevisiae*, which is a subunit of the HIRA complex and is involved in replication-independent histone deposition [17], [18]. It is known that the HIRA complex is evolutionally conserved from yeast to humans, and that it plays important roles in position-dependent gene silencing and nucleosome reassembly [26], [27]. In *S. pombe*, the HIRA complex is composed of four subunits: Hip1, Slm9, Hip3 and Hip4 [17], [27], [28]. Crystal structure analysis revealed that the B domain of Hip1 directly binds to another histone chaperone, spAsf1 (Cia1) [21]. In addition, deletion mutants of each HIRA subunit exhibit impaired transcriptional silencing of centromeric heterochromatin and repression of the *Tf2* retrotransposon [27]. We identified a *hip3-1* mutant by its defects in the silencing of the meiosis-specific gene *SPCC663.14c* ^+^, which is predicted to encode a TRP-like ion channel protein and is repressed in rich media containing nitrogen.

We also identified the *nrd1^+^* gene as a multicopy suppressor of *hip3-1* mutation (Fig. 3A). Nrd1 is involved in repressing Ste11-mediated transcription [17]. Because the *nrd1^+^* gene suppresses the *ts^-^* phenotype of *hip3-1* mutant, it is possible that the transcription of meiosis-specific genes affects the growth of *hip3-1* mutant cells at restrictive temperatures. However, the overexpression of Nrd1 did not completely rescue the *ts^−^* phenotype of *hip3-1* mutant (Fig. 3A), suggesting that this *ts^−^* phenotype is not caused simply by the inhibition of the transcriptional repression of meiosis-specific genes.

In *hip3-1* cells, the *SPCC663.14c* ^+^ gene was found to be expressed under rich conditions (Fig. 5). However, the *ura4^+^* gene was not expressed in *hip3-1* cells that were transformed with pSP1-ste6URA4, whose promoter was replaced with the promoter of another meiosis-specific gene, *ste6^+^,* which functions as a guanine-exchange factor (GEF) and is activated by Ste11 (Fig. 3D) [29]. In addition, it has been reported that the deletion of the other HIRA subunits affects the transcription levels of some (but not all) meiosis-specific genes [26]. From their previous assay, the *SPCC663.14c* ^+^ gene was not included in the list of affected meiosis-specific genes. These data clearly indicate that not all meiosis-specific genes are transcriptionally repressed by the HIRA complex.

ChIP analyses revealed that the occupancy of histone H3 was reduced at the *adh1* ^+^ and *SPCC663.14c* ^+^ loci but not at the *adh1* ^+^ promoter in *hip3-1* cells (Fig. 6C). Because the level of histone H3 was much lower at the *adh1* ^+^ promoter than at other loci in wild-type cells, the histone H3 levels might not be significantly different between wild-type and *hip3-1* cells at the *adh1* ^+^ promoter. However, in *hip3-1* cells, the extent of histone modification was completely distinct from that in wild-type cells (Fig. 7). Acetylated H3K9 accumulated in the intragenic region of the *adh1* ^+^ locus in wild-type cells, whereas histone H3 was rarely acetylated at the *SPCC663.14c* ^+^ locus. Because it is known that H3K9 acetylation correlates with transcriptional activation, this suggests that the difference in the number of acetylated H3K9 residues between the *adh1* ^+^ and *SPCC663.14c* ^+^ loci is caused by a difference in their transcription levels. By contrast, tri-methylated H3K9 was enriched at the *SPCC663.14c* ^+^ locus compared to the *adh1* ^+^ locus (Fig. 7B), suggesting that there is an inverse correlation between acetylation and tri-methylation of H3K9. However, this is not the case for *hip3-1* mutant. Several modifications of histone H3 occurred extensively at the *SPCC663.14c* ^+^ locus in *hip3-1* cells. Notably, the tri-methylation of H3K9 and H3K4 were enriched at both the *adh1* ^+^ and *SPCC663.14c* ^+^ loci (Fig. 7), and these modifications occurred widely in *hip3-1* mutant (Fig. 8). In budding yeast, the histone chaperone Asf1p is required for histone H3 lysine 56 (H3K56) acetylation [30]. In fission yeast, the histone deacetylase (HDAC) Clr6 is involved in the global deacetylation of histones and associates with the fission yeast HP1 homolog protein Swi6 [31], [32]. It was recently reported that the HIRA complex associates with Swi6 and facilitates global histone deacetylation [33]. In this study, the level of histone H3K9 acetylation was not elevated in *hip3-1* mutant (Fig. 8). This difference is probably caused by the particular mutation in *hip3-1* mutant. The Hip3-1 protein is truncated at the C-terminus of Hip3 and can interact with spASF1 (Fig. 4). In addition, ASF1 associates with the H3K4 demethylase LID and is required for the targeted removal of the positive H3K4me3 mark by facilitating the recruitment of LID to chromatin in *Drosophila* [34]. Histone chaperones are required for proper histone modifications in many organisms. In *hip3-1* cells, the levels of *SPCC663.14c* ^+^ mRNA were about 1.4-fold higher than in wild-type cells (Fig. 5), and a GFP-NLS fusion protein was expressed in *hip3-1* cells that were transfected with pSP1-meiGFP-NLS (Fig. 3D), suggesting that the HIRA complex is involved in achieving appropriate histone modifications and transcriptional repression. We are currently thinking that the reason why the effect of Hip3 is such mild might be because some other critical factors are involved together with Hip3 but could not be detected because of their lethality. In addition, Pol II and the general transcription factor TFIIE were concentrated at the *SPCC663.14c* ^+^ promoter region in *hip3-1* cells (Fig. 6A and B). TFIIE is known as one of five general transcription factors (GTFs) that are essential for transcriptional initiation and the transition from initiation to elongation [35]. GTFs form a PIC with Pol II and regulate the transcription levels of genes. The enrichment of spTFIIE and Pol II at a meiosis-specific gene promoter suggests that aberrant histone modifications affect transcriptional regulation and the efficiency of PIC formation by GTFs and Pol II.

It is well known that tri-methylated histone H3K9 recruits HP1 and is involved in heterochromatin formation [8]. However, the levels of trimethylated H3K9 were found to be elevated in *hip3-1* cells (Fig. 8), and transcription occurred at the region that was enriched with trimethylated H3K9 (Fig. 6). The discrepancy between these phenomena in the *hip3-1* mutant might be caused by “scrambled heterochromatin factors.” Because heterochromatin factors are recruited to chromatin that contains tri-methylated H3K9, excess tri-methylation of H3K9 could result in a shortage of these heterochromatin factors at their proper positions. Transcriptional repression might be inhibited in this way in *hip3-1* mutants.

Our data indicate that the HIRA complex is required for nucleosome assembly and the transcriptional repression of meiosis-specific genes through the regulation of histone modification. However, the HIRA complex's regulatory mechanisms are not fully understood. It is possible that chromatin structure controls the interactions between core histones and their modifications. From our data, we speculate that our identified hip3-1 mutation causes aberrant chromatin organization which triggers disruption of chromatin structure. And, finally, abnormal histone modifications will be emerged. We think that the levels of gene transcription were determined by the final valance of positive and negative effects on each gene transcription. Therefore, we also think that we will not be able to judge the extent of transcription activity simply by the alteration of individual histone mark. To date, many studies have reported that histone modifications regulate both chromatin structure and transcription. Our results will provide a new clue regarding how chromatin structure and histone modifications influence each other and determine the transcription level.

## Materials and Methods

### Yeast strains, media and the general method

The *S. pombe* strains used in this study are shown in Table 1.

**Table 1:** *S. pombe* strains used in this study.

Strain	Genotype	source
UR471	*h^−^, leu1-32, ura4-D18*	[36]
*hip3-1*	*h^−^, leu1-32, ura4-D18, hip3-1*	This study
*Hip3-flag*	*h^−^, leu1-32, ura4-D18, Hip3-flag (KanMX6)*	This study
*hip3-1-flag*	*h^−^, leu1-32, ura4-D18, hip3-1-flag (kanMX6)*	This study

Complete medium (YE) supplemented with 80 mg/liter adenine and leucine (YEAL), minimal medium (MM) and nitrogen-depleted medium (MM-N) were used for the standard cultures. Appropriate growth supplements (80 mg/liter adenine, leucine, and uracil) were also added to the MM. SPA medium was used for the induction of mating and sporulation of *S. pombe*. Medium containing 2% agar was used for plating. The standard genetic methods for *S. pombe* were as described previously [37], [38].

### Construction of a reporter plasmid for the transcriptional repression assay

To construct a reporter plasmid to detect transcriptional repression, *Sal*I and *Pst*I sites were created at the 5′ and 3′ ends, respectively, of the intergenic region between SPCC663.14c and SPCC663.15c by PCR from *S. pombe* genomic DNA. Similarly, *Pst*I and *Bam*HI sites were added to the 5′ and 3′ ends, respectively, of the *ura4^+^* open reading frame, which also encodes the GFP-SV40 NLS fusion protein from the phrGFP-Nuc plasmid (Stratagene). These fragments were digested with *Sal*I and *Pst*I or with *Pst*I and *Bam*HI and ligated into a pSP1 vector that had been digested with *Sal*I and *Bam*HI.

### RNA isolation and quantitative RT-PCR

Wild-type and *hip3-1* cells were grown in 10 ml of YEAL medium at 30°C until mid-log phase. The cells were washed twice with sterilized DDW, and their total RNA was prepared using glass-bead disruption as described by Urushiyama *et al.* [39]. The total RNA yield was usually 40–200 µg.

The RNA samples were treated with RNase-free DNase I in 8 mM MgCl_2_ and 40 mM Tris-HCl (pH 7.5) at 37°C for 30 min to remove contaminated genomic DNA, extracted with phenol/chloroform/isoamylalcohol (98:1:1) and precipitated with ethanol. Reverse transcription was then performed using the PrimeScript II 1st strand cDNA Synthesis Kit (2.5 µM oligo-dT primer and 2 µg of total RNA) according to the manufacturer's protocol. Real-time PCR was performed in a solution containing SYBR Premix Extaq II (Takara), 4 µM of primers for *adh1* ^+^ or *SPCC663.14c* ^+^ mRNA, and 0.5 µl of reverse-transcribed cDNAs.

### Chromatin immunoprecipitation

Chromatin immunoprecipitation (ChIP) was performed essentially as described previously but with minor modifications [25]. A 50-mL volume of UR471 (wild-type) and *hip3-1* cells were grown to an optical density of 0.9 at 600 nm in YEA medium. Formaldehyde was added at a final concentration of 1% for 30 min at room temperature, and incubation continued with 120 mM glycine at room temperature for 5 min. The cells were harvested by centrifugation, washed twice with chilled Tris-buffered saline, and treated with Zymolyase 100T (Seikagaku Biobusiness) and lysing enzymes from *Trichoderma harzianum* (Sigma) in cell-wall-digestion buffer (50 mM HEPES-KOH (pH 7.5), 10 mM MgCl_2_, 1 mM EGTA, 1.2 M sorbitol). The cells were incubated at 30°C until the cell wall was digested, as determined by phase contrast microscopy. After washing with cell wall-digestion buffer, the cells were dissolved in lysis buffer (50 mM HEPES-KOH (pH 7.5), 140 mM NaCl, 1 mM EDTA, 1% Triton X-100, 0.1% Na-deoxycholate, 1 mM PMSF, 1 µg/mL leupeptin, 1 µg/mL pepstatin A). The cells were frozen and homogenized with a mortar and pestle under liquid nitrogen. The chromatin was sonicated to yield fragments ranging from 200 to 800 bp using a Covaris^TM^ S2 system. This cell lysate was mixed with the antibody at 4°C for 12 hr and incubated in a Protein G-Sepharose 4 Fast Flow (GE Healthcare) at 4°C for 2 hr. The resin was then collected by centrifugation and successively washed with lysis buffers containing 140, 250 and 500 mM NaCl. After washing with LiCl/detergent wash buffer (1 mM LiCl, 10 mM Tris-HCl (pH 7.9 at 4°C), 1 mM EDTA, 0.5% Na-deoxycholate, 0.5% Nonidet P-40) followed by TE buffer (pH 8.0), the resin was incubated with elution buffer (50 mM Tris-HCl (pH 7.9 at 4°C), 10 mM EDTA, 1% SDS) for 10 min at 65°C. The ChIP fractions were collected by centrifugation and treated with 400 mg/ml proteinase K, and the cross-linking was reversed by incubating at 65°C for 12 hr. Finally, real-time PCR was performed using SYBR Premix Extaq II (Takara) to quantitate the fragments.

### Co-immunoprecipitation (Co-IP)

To tag the chromosomal *hip3^+^* genes, we constructed DNA fragments that were amplified by PCR using two pairs of primers: hip3-13 and hip3-wt-tag-N3 and hip3-tag-C5 and hip3-15. Using the hip3-13 and hip3-15 primers, two fragments and *Eag*I-digested pFA6a-flag *KanMX6* ^+^ as templates, a PCR reaction was performed. The PCR products were transfected into UR471 cells and selected on YEAL plates containing G418. The tagged Hip3- or Hip3-1-expressing strains were confirmed by PCR. These strains were transformed using the pREP42-HA-Asf1 plasmid, which expresses 1xHA-tagged spASF1/Cia1 driven by the *nmt1* promoter. The transformants were incubated at 30°C until mid-log phase and harvested by centrifugation. The cells were dissolved in the same volume of Buffer B (20 mM Tris-HCl (pH 7.9), 10% glycerol, 1 mM phenylmethylsulfonyl fluoride (PMSF), 2 µg/mL antipain, 2 µg/mL aprotinin, 1 µg/mL leupeptin, 0.8 µg/mL pepstatin, 10 mM 2-mercaptoethanol) containing 150 mM NaCl. The cells were then mixed with the same volume of glass beads and disrupted by vortexing. The cell lysates were immunoprecipitated for 2 hr at 4°C with anti-FLAG M2 agarose (Sigma-Aldrich). The pellets were washed four times with Buffer B containing 200 mM NaCl, subjected to SDS-PAGE, and blotted onto a nylon membrane. HA-tagged spAsf1/Cia1 was detected using an anti-HA antibody (3F10), an HRP-conjugated anti-rat antibody, and ECL Plus (GE Healthcare).
